# A Gold Nanoparticle Bouquet held on plasma membrane: An ultrasensitive dark-field imaging approach for Cancer Cell Analysis

**DOI:** 10.7150/ntno.41639

**Published:** 2020-06-20

**Authors:** Yue Cao, Jie Wang, Qiao-Yan Jiang, Li Hu, You-Jia Yu, Yan-Fang Yu, Feng Chen

**Affiliations:** Department of Forensic Medicine, Nanjing Medical University, Nanjing, Jiangsu, 211166, PR China.

**Keywords:** gold nanoparticle bouquet, dark-field imaging, p53, cancer cell, photothermal therapy

## Abstract

**Rational:** p53 is suppressing tumor protein correlated with the cell cycle factors and apoptosis. Here, a gold nanoparticle bouquet is designed for an ultrasensitive dark-field imaging approach for cancer cell analysis.

**Methods:** AuNP60/APBA is functionalized by a gold nanoparticle bouquet-plasmonic 60 nm gold nanoparticles. And consistent APBA can be held on the plasma membrane. After 13 nm gold nanoparticles are functionalized with mannose (AuNP13/MN), the AuNP60/APBA gold nanoparticles are captured. The absorption spectrum of aggregation gold nanoparticles (AuNPs) shifts to near-infrared (NIR) region which can be observed under dark-field microscopy (DFM) and is treated the subsequent with photothermal therapy.

**Results:** The results that MCF-7 cells were successfully destroyed under the near-infrared (NIR) irradiation and the intracellular WTp53 increased while the MTp53 decreased. These results indicated that p53 is the key molecule in the apoptosis signaling pathway. Photothermal therapy can stimulate the MTp53 in the cell signal conductive pathway.

**Conclusion:** This work offers a new method for intracellular p53 analysis and a potential targeted cancer treatment.

## Introduction

Noble metal nanoparticles, peculiarly gold nanoparticles (AuNPs), keep attracting much attention due to its excellent biocompatibility and picturesque optical properties 1-5. AuNP is an attractive nanomaterial candidate with resonant absorption and excitation of incident light to induce strong local surface plasmon resonance (LSPR) 6,7. With the continuous development of dark-field hyperspectral imaging (DFM) and plasmon resonance scattering spectrum, the impact of plasmonic nanoparticles has been successfully applied in biosensing 6, single particle analysis 8, colorimetric biosensors 9, and single-cell monitoring 10. On one hand, AuNPs has a strong absorption capacity and high heat conversion efficiency, which was also inspired by the AuNPs' plasma properties and excellent tolerability 11. Although AuNP-based light therapies for cancer have been widely reported 12-14, these methods lack the ability to do cell surface photothermal therapy or track cancer marker p53. In this field, it's worth emphasizing that how flexibility to design new-style AuNP-based photothermal reagents for high sensitivity and selectivity of cancer therapy. On the other hand, taken the clinical trials consideration the clinical trials, it is expected that the photothermal emission of absorption of long-wave light in the near-infrared (NIR) region can further penetrate the tissue 15,16. To accomplish this target, AuNPs of varying forms and sizes have been prepared for NIR illumination and attained a good therapeutic effect 17. Regrettably, these nanoparticles cannot fixedly aggregate on the cell membrane since they can easily be endocytosed into cells. This prompted us to design an intelligent system that assembled on the surface of cancer cells after the small-sized AuNPs (The diameter of which is less than 20 nm) capture the 60 nm gold which is immobilized on the cell membrane. The system can form a gold nanobeam, whose absorbing light is in the near-infrared region, for subsequent photothermal therapy.

Human p53 is a tumor-suppressing protein to controls cell dividing cycle and apoptosis (programmed cell death) by controlling tumor progression 18. There is a central specific DNA-binding domain in present human WTp53 (wild-type p53 protein) 19. The DNA-binding center region of p53 protein loses binding capacity due to extensive mutation in p53 gene in various types of cancer cells 20. Abnormal expression of MTp53 (mutant-type p53 protein) can serve as an important carcinogenesis stimulus 21. Thus far, various methods for detecting endogenous p53 have been reported, including enzyme-linked immunosorbent assay (ELISA) 22, immunohistochemically assay 23, electrophoretic mobility shift assay 24, and ds-DNA consensus binding biosensing assay 25. Despite the favorable detection limit and sensitivity of these methods, none of these methods linked at the relationship between photothermal therapy and p53 protein signaling pathways. Hence, it is urgently needed to design *in situ* analysis strategies for photothermal therapy of cell membrane surface to analyzing the changes of p53 protein in cells.

**Figure 1:**
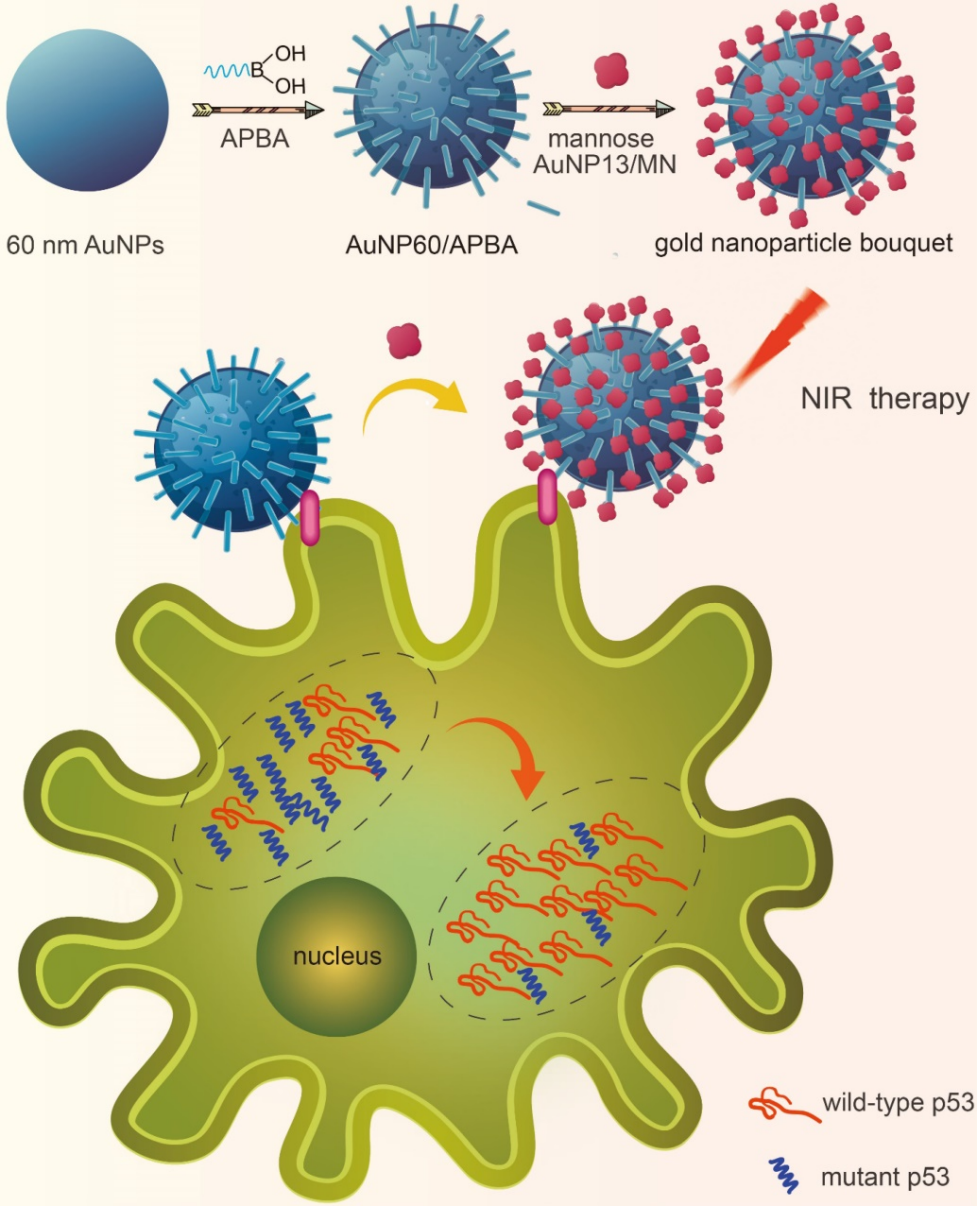
A schematic illustration of a gold nanoparticle bouquet (AuNP60/APBA-AuNP13/MN) for *in situ* analysis of in-tracellular WTp53 and MTp53.

To investigate whether the gold nanoparticle bouquet can downregulate MTp53 during photothermal therapy, the gold nanoparticle bouquet (AuNP60/APBA-AuNP13/MN) was designed through dual functionalization of a single 3-aminophenyl boronic acid (APBA) assembled on the surface of gold nanoparticles (AuNP60) with amine-gold links to construct AuNP60/APBA. APBA binds to mannan-conjugated gold nanoparticles (AuNP13/MN) 26,27. The AuNP60/APBA, which is a plasmonic AuNP, can held on the plasma membrane through the competitive reaction of cell surface SA with AuNP13/MN. Thus SA can replace AuNP13/MN and bound to AuNP60/APBA. AuNPs absorption can be transferred to near-infrared by polymerization. They can not only can be viewed under dark-field hyperspectral imaging (DFM), but also be used for the subsequent photothermal therapy. It is showed that MCF-7 cells were successfully broke under-near-infrared (NIR) irradiation. And the intracellular WTp53 increased while the MTp53 decreased (Figure 1). These results indicated that p53 is the key molecule in the apoptosis signaling pathway. Photothermal therapy can stimulate the MTp53 in the cell signal conductive pathway. This research affords a new strategy for studying intracellular p53 analysis through photothermal therapy on the cell surface and finds a potential *in-situ* treatment against cancer.

## Experimental Section

### Sample Preparation for the Detection with Dark-Field Microscopy (DFM) and Scattering Spectroscopy

The AuNPs that were used for DFM images were fixed on the surface of the glass sheet. The glass sheet was sonicated in ethanol and then washed with pure water. The clean glass sheet was dried, and the AuNP solution was deposited on the surface to evaluate the DFM detection. To image cells that had absorbed AuNPs, the cell suspension (1 mL, 1×10^6^ mL^-1^) was seeded in each culture dish (the bottom is glass sheet) and cultured overnight. Then, 30 μL of the AuNP-60/APBA was added to each dish and incubated for various times at 37 °C. Furthermore, the dishes were washed twice and immersed in PBS for DFM imaging. Then, 50 μL of the AuNP-wrapped mannan was added to each dish. The AuNP-scattered light was split using a grating, and the scattering spectra of the samples were recorded by CCD spectrometer.

Supporting Information (Experimental Section).

## Result and Discussion

### Characterization of the gold nanoparticle bouquet

**Figure 2:**
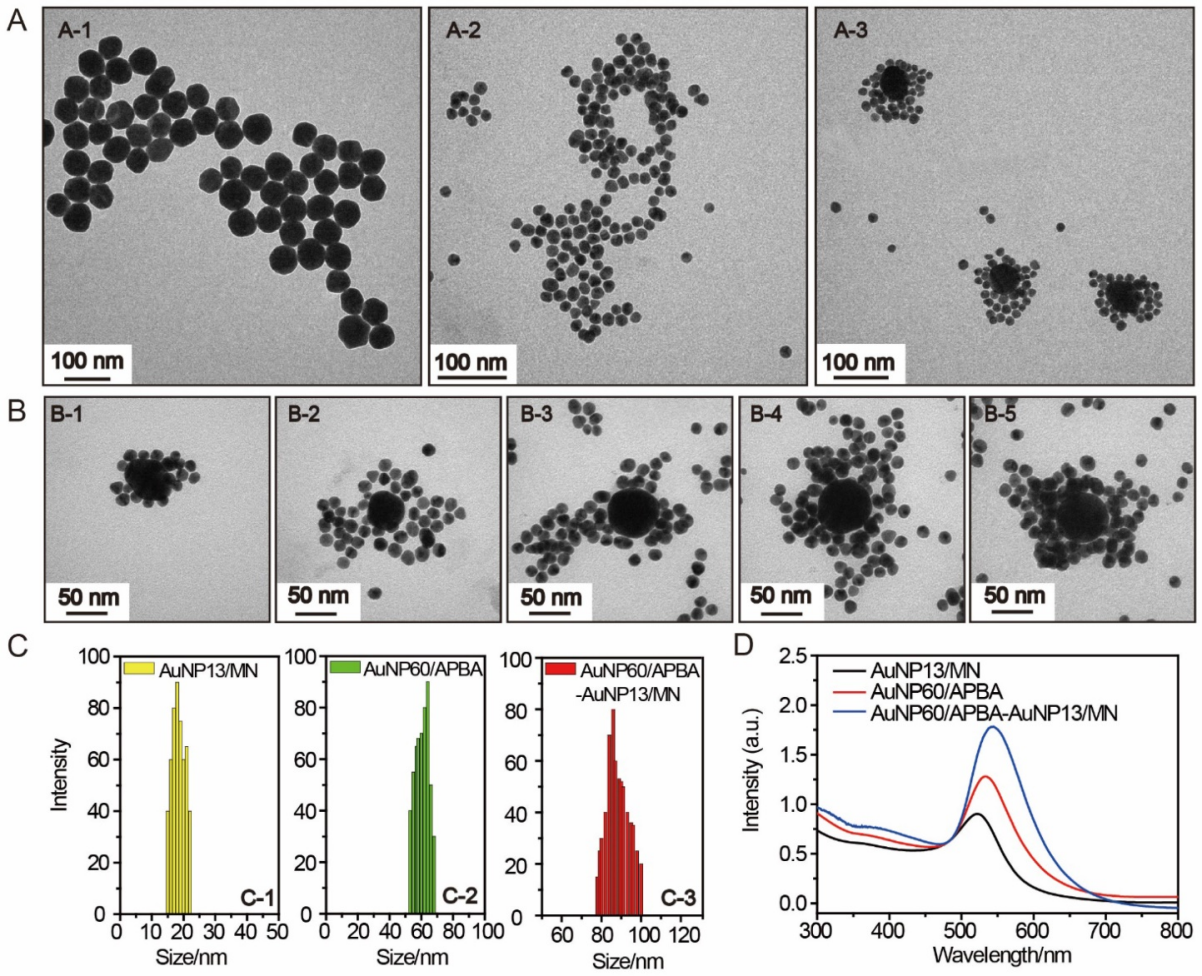
(A) TEM image of the prepared AuNP60/APBA (A-1), AuNP13/MN (A-2) and AuNP60/APBA-AuNP13/MN (A-3). (B) TEM images of the AuNP60/APBA incubated with AuNP13/MN at different concentration (final AuNP13/MN concentration: 2, 4, 6, 8 and 10 nM from (B-1) to (B-5). (C) Dynamic light scattering (DLS) result of the prepared AuNP13/MN, AuNP60/APBA and AuNP60/APBA-AuNP13/MN. (D) UV-Vis spectra of AuNP13/MN, AuNP60/APBA and AuNP60/APBA-AuNP13/MN.

**Figure 3:**
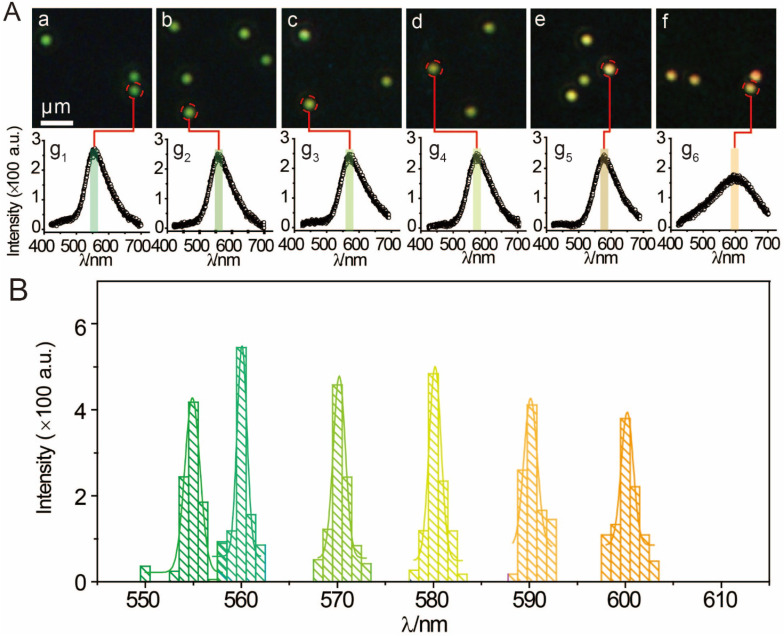
(A) DFM images of 30 μL AuNP60/APBA incubated with 50 μL AuNP13/MN for 100 min with different concentrations (final AuNP13/MN concentration: 0, 2, 4, 6, 8 and 10 nM from (a) to (f). g1)-g6): Corresponding scattering spectra of AuNP60/APBA observed in DFM images. (B) Corresponding statistical graph of AuNP60/APBA in DFM images a-f in (A).

**Figure 4:**
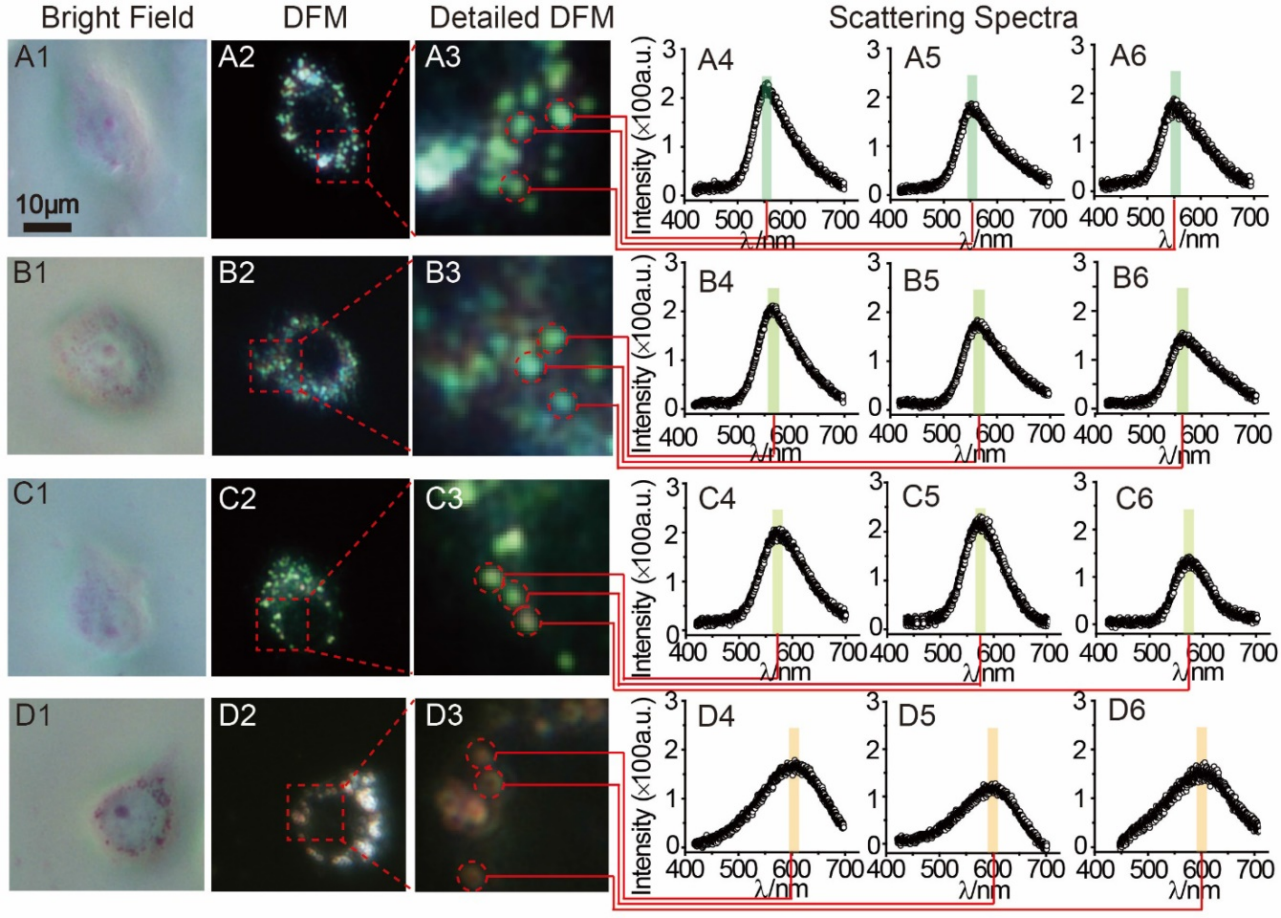
DFM images of 30 μL AuNP60/APBA incubated with a single MCF-7 cell (treated with 0, 2, 5 and 10 nM) AuNP13/MN from A to D) for 100 min. (A1-D1: bright field; A2-D2: DFM images; A3-D3: Detailed DFM; A4-6-D4-6: corresponding scattering spectra).

**Figure 5:**
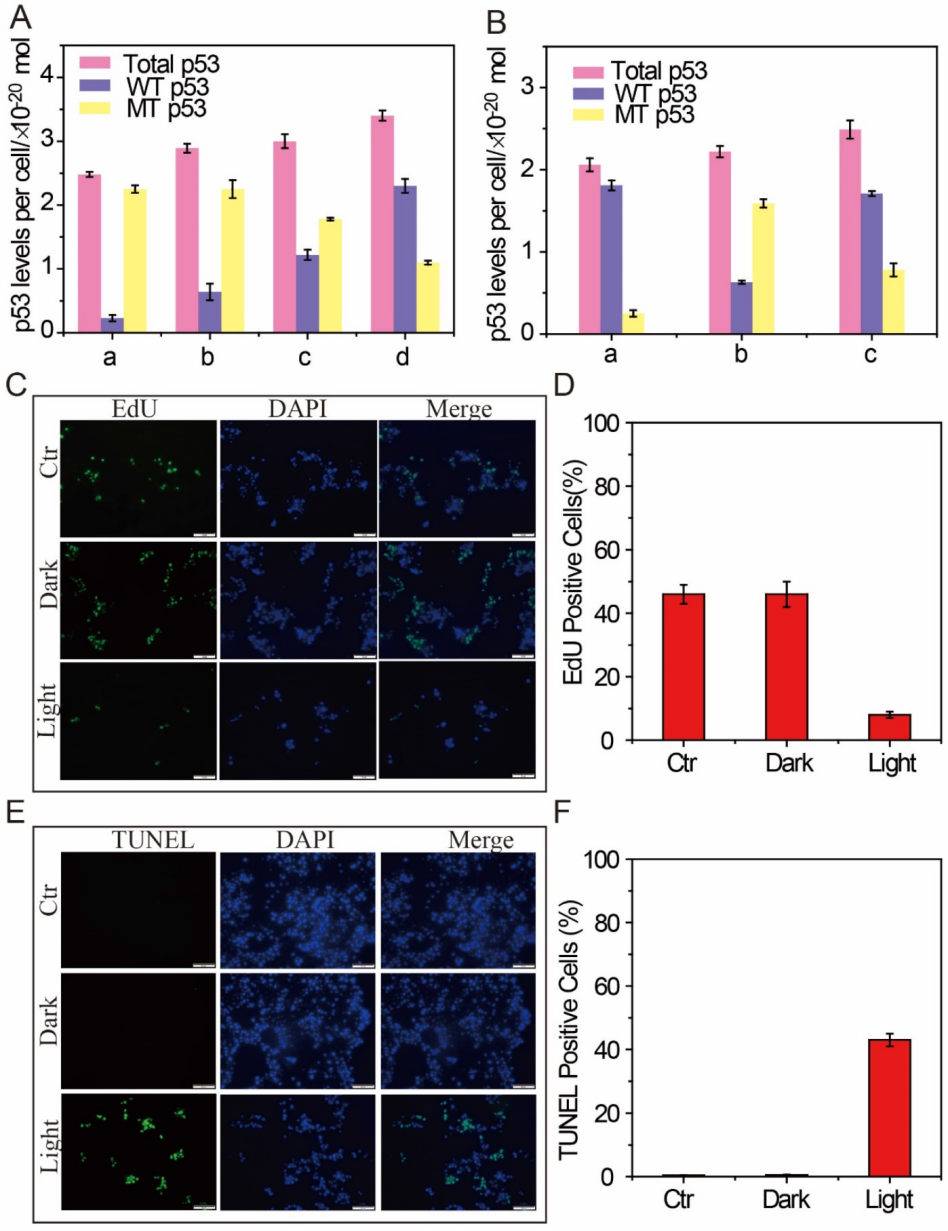
(A) p53 levels (total p53, WTp53 and MTp53) in MCF-7 cell (detected from cell extracts collected from MCF-7 cells treated with: AuNP60/APBA contain (no, 2, 5, 10 nm) AuNP13/MN from A to D. (B) p53 levels (total p53, WTp53 and MTp53) in (A: HBE cells; B: MCF-7 cells; C: Added AuNP60/APBA-AuNP13/MN nanoparticle bouquet to MCF-7 cells. (C) EdU incorporation assay in MCF-7 cells. Scale bars = 50 μm. (D) Quantitative measurement of EdU positive cells showed in C. (E) TUNEL assay in MCF-7 cells. Scale bars = 50μm. (F) Quantitative measurement of TUNEL positive cells showed in E. Results are representative of at least 3 separate experiments and expressed as mean ± SE.

The preparation of 60 nm AuNPs is by the method previously reported 28. The AuNPs were modified APBA and could identify the sialic acid SA site. The mannan-conjugated 13 nm AuNPs were synthesized through a one-pot synthetic process with mannose-polysaccharide mannan as stabilizer and NaBH4 as the reducing reagent.^29^. The image of AuNP60/APBA was taken by transmission electron microscopy (TEM). We can see from Figure 2A-1 that the average size of the AuNP60/APBA nanoparticles is 60 nm and it distributes evenly. Figure 2A-2 indicates that the diameter of the AuNP13/MN diameter is 18 nm, which is consistent with the dynamic light scattering (DLS) result of 18 nm and is 5 nm larger than the naked AuNPs synthesized under the same condition without the presence of mannan (Figure 2C). TEM images of the AuNP60/APBA incubated with AuNP13/MN at different concentrations were shown in Figure 2 A-3, Figure 2B, B-1, B-2, B-3, B-4, B-5. The AuNP13/MN, AuNP60/APBA and AuNP60/APBA-AuNP13/MN nanoparticles presented a negative zeta potential (Figure S1). As shown in Supporting Information Figure S2, green spots (AuNPs) were observed from the DFM image. The modified AuNP60/APBA still has green spots, which means that the diameters of the AuNP60/APBA and the bald AuNPs are the same. The UV-Vis spectrum of the AuNP60/APBA showed a characteristic peak at 535 nm, which red shifted to 548 nm after added AuNP13/MN (522 nm) was conjugated on their surface, which demonstrated the successful connection of the AuNP13/MN and AuNP60/APBA (Figure 2D). The average amount of APBA assembled on each nanoparticles was measured to be around 1.44×10^6^. The concentration-dependent temperature increase of AuNP60/APBA-AuNP13/MN nanoparticles bouquent was detected with the extension of laser illumination time, as indicated in Figure S3A. After 50 min of irradiation, the temperature of AuNP60/APBA-AuNP13/MN aqueous solution was increased to 42°C at the maximum concentration of 10 nm (Figure S3B). Then, the photothermal conversion efficiency (η) of AuNP60/APBA-AuNP13/MN nanoparticles bouquent exposed to 680 nm lasers for 500 s was performed and the photothermal conversion efficiency value of AuNP60/APBA-AuNP13/MN nanoparticles bouquent was calculated to be 40.6%.^30^ (The detailed process is provided in the Supporting Information.

### *In situ* Dark Imaging of the gold nanoparticle bouquet

To optimize the incubation time for the binding reaction between AuNP60/APBA and AuNP13/MN in solution, the DFM images were recorded at different time points after a series of AuNP13/MN solution (50 μL) were incubated with AuNP60/APBA (30 μL, 5 μM) at 37°C. As shown in Supporting Information Figure S4, the color of AuNPs by degrees changed from green to orange and stopped changing after 100 min, which demonstrated the aggregation of AuNP13/MN and AuNP60/APBA-triggered. A series of different concentrations of AuNP13/MN solutions were incubated with AuNP60/APBA (30 μL, 5 μM) solution according to the optimum reaction time. Figure 3A demonstrated this by the fact that the color was green at a low concentration and the peak of the scattering spectrum was basically the same as that of the control group (Figure 3Aa, g1). The interaction between the AuNP60/APBA and the increase of AuNP13/MN concentration caused a dramatic color change to yellow and then orange and the scattering peak shifted from 550 nm to 610 nm upon the AuNP13/MN and AuNP60/APBA conjugation (Figure 3A b-f, g2-g6). Therefore, we developed an expedient quantification approach for AuNP60/APBA DFM images processed with AuNP13/MN at different concentrations. Corresponding statistical scattering spectral peak of the AuNP60/APBA was determined using the Gaussian function (Figure 3B). The distribution histogram showed the relationship between the AuNP60/APBA and different concentrations of the AuNP13/MN.

### *In vitro* Studies

MCF-7 cells (1 mL, 1×10^6^ mol∙L^-1^) were planted in a petri dish for intracellular testing. After the adding of 30 μL AuNP60/APBA, DFM images were obtained at different time points. Only a small amount of AuNP60/APBA particles were observed in the first 30 min. After 1 h, significantly more AuNP60/APBA nanoparticles appeared on cell membrane surface. The number of AuNP60/APBA increased with the extended incubation time until reached the plateau after 2 h (Supporting Information, Figure S5). MCF-7 cells incubated with different concentrations of AuNP13/MN were analyzed by DFM imaging and scattering spectroscopy at 2h optimum incubation time (Figure 4). From Figure 4 A1-A3, the AuNP60/APBA nanoparticles bouquet had a green color and has a peak scattering spectrum at approximately 520 nm (Figure 4, A4-A6), which indicated no AuNP13/MN in the MCF-7 cells. However, with increasing doses of AuNP13/MN, the color of AuNP60/APBA nanoparticle bouquet became orange in the DFM images (Figure 4, B3, C3, D3), and the scattering peak gradually redshifted from 520 nm to 610 nm little by little (Figure 4, B4-B6; C4-C6; D4-D6), after incubation with different concentration of the AuNP13/MN for 2 h, these changes indicated that the formation of AuNP60/APBA nanoparticle bouquet depends on the dose-dependent increase of AuNP13/MN in intracellular.

In addition, after the intracellular DFM images, the feasibility of p53 apoptotic signalling pathway based on the AuNP60/APBA nanoparticle bouquet was applied by MCF-7 cell-free extracts. The change of p53 level in MCF-7 cells was studied by using AuNP60/APBA nanoparticle bouquet as a simulation drug through NIR irradiation. After incubated with AuNP60/APBA for 2 h, the MCF-7 cells (1 mL, 1×10^6^ mL^-1)^ extracts were added with different amounts of AuNP13/MN solution for 12h. A p53 pan ELISA kit (KeyGen Biotech. Co. Ltd., Nanjing, China) was used to detect the level of total p53 (WTp53 and MTp53), extraction of the above cell extracts for testing and establishment of the standard calibration curve (Supporting Information, Figure S6, Figure S7). After drug treatment, the total p53 level was increased, while the MTp53 level gradually declined (Figure 5A), which indicated through NIR irradiation of AuNP60/APBA-AuNP13/MN nanoparticle bouquet ability to stimulate the MTp53 in cell signal conductive pathway.

To demonstrate that photothermal therapy using AuNP60/APBA-AuNP13/MN nanoparticle bouquet could adjust MTp53 content, WTp53 and MTp53 in the MCF-7 cell extracts were collected respectively for measurement after the therapy. The result showed that the expression of WTp53 was upregulated while the expression of MTp53 was downregulated. The identification of WTp53 and MTp53 in HBE cells, a non-tumor cell line, was used as control-experiment (Figure 5B). P53 levels were normal in the HBE cells, but MTp53 levels were abnormal in MCF-7 cells. At the same time, when the AuNP60/APBA-AuNP13/MN nanoparticle bouquet was added to MCF-7 cells for photothermal treatment, the MTp53 levels have been decreased by a half and the number of WTp53 increased by one times (Figure 5B). The results indicate that photothermal therapy can stimulate the MTp53 in the cell signal conductive pathway and the cells enter normal apoptosis state.

The above results have successfully proved that the proposed binary system can efficiently bind AuNP probe on the surface of living cells to form a polymer in the presence of AuNP13/MN. MCF-7 cells were used as a model to investigate whether the aggregates could be applied to kill tumor cells under NIR photothermal therapy. After incubation with the different concentrations of AuNP60/APBA nanoparticle bouquet for 12 h, the cells were irradiated with a laser (680 nm, 0.5 W cm^-2^, 37 °C). The absorbances were examined to ascertain the relative cell activity. Cell viability remained at 90 % in the dark (Supporting Information, Figure S8), suggesting that AuNP60/APBA nanoparticle bouquet were biocompatible in cell experiments. However, after 20 min of NIR irradiation, morphological changes of cells (Figure S9) and the apoptotic MCF-7 cells bursted, which demonstrated that AuNP60/APBA nanoparticle bundles were capable of killing cancer cells under NIR illumination in a cell biocompatibility assay system (Figure S8). Meanwhile, consistent with the results from the MTT assay, EdU incorporation assay (Figure 5C and 5D) and TUNEL assay (Figure 5E and 5F) revealed that the proliferation of MCF-7 cells was reduced while the apoptosis was increased in light group. TEM images showed the AuNP60/APBA-AuNP13/MN nanoparticle bouquet in MCF-7 cell surface (Figure S10). Overall, these results revealed that AuNP60/APBA-AuNP13/MN nanoparticle bouquet combined with illumination showing an excellent anti-tumor effect on human MCF cells.

### *In vivo* Studies

**Figure 6:**
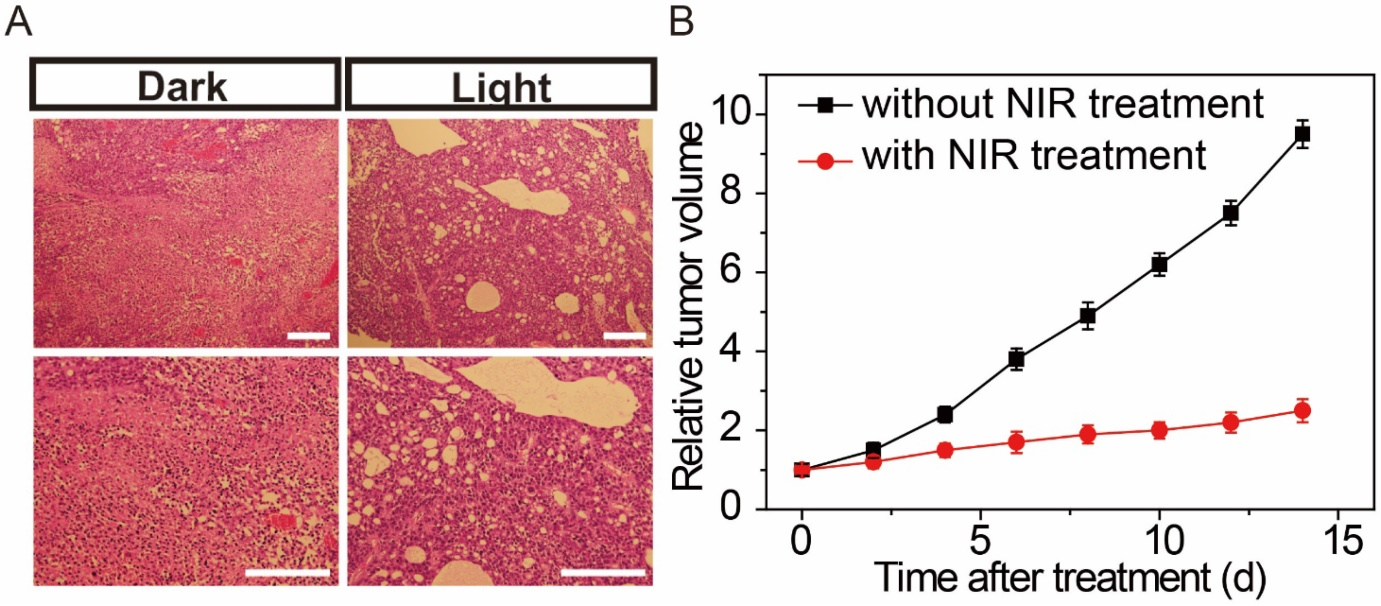
(A) Histological observation of the tumor tissues without and with NIR treatment hypodermic injection of the gold nanoparticle bouquet after 7 days. (B) Change of tumor volume with or without NIR treatment for 2 weeks. Scale bars: 200 μm.

On the basis of former cell NIR studies, we further established a binary system to monitor the effects of NIR therapeutic in living mice. Feminine pathogen free (5-6 weeks) BALB/c nude mice were used as tumor models. Under isoflurane anesthesia, MCF-7 cells in the logarithmic growth stage were injected subcutaneously into the flanks of the nude mice. Then, the AuNP60/APBA-AuNP13/MN nanoparticle bouquet solution was hypodermically injected into the tumor-bearing mice. The nanoparticle bouquet could efficiently arrival the tumor region, mice with the MCF-7 tumor were treated with a NIR laser (power of 0.5 W cm^-2^) irradiation for 30 min to treatment, while mice without NIR treatment were served for control. As shown in Figure 6, after 7 days of NIR treatment, the tumor status was monitored. Histological examination was carried out to observe the tumor tissues. The tumor tissue sections were clearly necrotic, while the control group showed no apparent cell destruction (Figure 6B). The results show that the proposed system NIR therapy has an obvious therapeutic effect on tumor tissue.

## Conclusion

In this work, we designed the AuNP60/APBA-AuNP13/MN nanoparticle bouquet to research the p53 in cells signal conductive pathway by photothermal therapy. The developed plasmonic AuNP60/APBA and AuNP13/MN sample can be easily prepared and had good biocompatibility. The AuNP60/APBA-wrapped AuNP13/MN readily entered living cells to do photothermal therapy. Evidently, the color changes are caused by the conjugation of AuNP60/APBA and AuNP13/MN, the formation of AuNP60/APBA-AuNP13/MN nanoparticle bouquet was observed, the changes of the p53 level in cells were studied through NIR irradiation. Thus, results showed that the intracellular WTp53 content increases while the MTp53 content decreases. The method is applicable to detect the changes of p53 level in cells during AuNP60/APBA-AuNP13/MN nanoparticle bouquet photothermal therapy, which is helpful to clarify the role of p53 in cell biological events. Thus, this capability demonstrates the effectiveness of the method for performing intracellular p53 analysis to target cancer therapy.

## Supplementary Material

Supplementary figures.
