# Supplementary material for: A Gold Nanoparticle Bouquet held on plasma membrane: An ultrasensitive dark-field imaging approach for Cancer Cell Analysis
Source: Nanotheranostics. 2020 Jun 20;4(4):201–9. doi: 10.7150/ntno.41639 (PMC7332797; doi:10.7150/ntno.41639)
Supplement: Supplementary file 1 — Supplementary figures. [file ntnov04p0201s1.pdf]

Supporting Information

A Gold Nanoparticle Bouquet Held on Plasma Membrane: an Ultrasensitive Dark-Field Imaging Approach for Cancer Cell Analysis

Yue Cao*, Jie Wang, Qiao-Yan Jiang, Li Hu, You-Jia Yu, Yan-Fang Yu, Feng Chen*

Department of Forensic Medicine, Nanjing Medical Univerisity, Nanjing, Jiangsu, 211166, PR China.

E-mail: ycao@nju.edu.cn; fchen@njmu.edu.cn

Materials and Reagents. All other reagents were of analytical grade. All aqueous solutions were prepared using ultrapure water (≥ 18 MΩ, Milli-Q, Millipore). Aminobenzeneboronic (APBA), chloroauric acid ($HAuCl_4$•$4H_2O$), mannose and bovine serum albumin (BSA) were obtained from Sigma-Aldrich (USA). Hydrochloric acid (HCl) and nitric acid (HNO_3) were purchased from Sinopharm. (Shanghai, China). P53 pan ELISA kit, anti-mutant p53 (anti-MTp53)/FITC, 3-(4,5-dimethylthiazol-2-yl)-2-diphenyltetrazolium bromide (MTT) was purchased from KeyGen Biotech. Co. Ltd. (Nanjing, China). MCF-7 cells was purchased ATCC. Phosphate buffer saline (PBS, pH 7.4) contained 2.7 mM KCl, 136.7 mM NaCl, 1.41 mM KH_2PO_4, and 8.72 mM Na_2HPO_4.

Apparatus. The transmission electron microscopic (TEM) images were obtained on a high-resolution transmission electron microscope (JEM-2010, JEOL Ltd., Japan). The dark-field images were viewed on an inverted microscope (IX73P2F, Olympus, Japan) containing a dark-field condenser (0.8 < NA < 0.95), a 40× objective lens (NA = 0.6). The size of the spot is determined by the objective. Since we used 40×objective, the size of spot should be around 500 μm. But our sample was smaller. The resolution of the instrument we used could reach up to 2 μm. The scattering light of the gold nanoparticle was split by a monochromator (HRS-300S, PI) equipped with a ruled grating (grating density 300 lines/mm with 750 nm blazed wavelength), and the scattering spectra were obtained by a spectrometer CCD (PIX-256E, Roper Scientific, PI). ELISA and MTT test were used on a microplate reader (Fluostar Omega, BMG Labtech, Germany). The NIR irradiation was executed by a 680 nm laser (Laserwave Optoelectronics Technology Co., Ltd., China).

Preparation of Gold Nanoparticles (60 nm AuNPs). All the glassware was dipped in aqua regia solution (HNO3/HCl, 1:3) for 12h (notice: aqua regia are highly corrosive and should be used with care) and then rinsed repeatedly several times with deionized water before use. Seed AuNPs with diameters of 13 nm were synthesized according to a procedure which has been described previously.[1] Put simply, HAuCl4 (0.01%, 50 mL) was added to a 100 mL round-bottomed flask that was fitted with a condenser. Under intense agitation, the solution was brought to a rolling boil and sodium citrate (5 mL, 38.8 mM) was rapidly added to the solution. The addition of the sodium citrate changed the color of the solution from pale yellow to red. Then, the solution was heated for 15 min. After the heating mantle had been stirred for an additional 15 min. The obtained solution of seed particles was applied to prepare the 60 nm gold particles using a method that has been described previously.[2] In brief, under vigorous stirring, seed particles solution (1 mL) and HAuCl4 solution (100 µL, 0.1%) were cautiously dropwise added into 25 mL of water within 5 min. The nanoparticle solutions were stored in brown bottles at 4 °C.

Preparation of ABPA Functionalized AuNPs (AuNP-60/APBA). First, 100 μL of ABPA (0.5 mg) was added to AuNP (1 mL) solution and the mixture was stirred overnight at room temperature. After that, was added PBS solution (0.1 mL) was added progressively to the mixture to gained the steady AuNP-60/APBA; the mixture was then centrifuged and washed with PBS twice and, finally, resuspended in 1 mL of PBS solution. The mixture solutions were stored in brown bottles at 4 °C.

Preparation of Mannan-conjugated Gold Nanoparticles (AuNP13/MN). The mannan-conjugated AuNPs were prepared by mixing a cooled solution (49.4 mL) containing 0.01% HAuCl4 and 3.2 mg mannan as the stabilizing reagent with 0.6 mL ice-cold solution of $NaBH_4$ (0.1 M) as the reducing agent[4] under continuously stirring, which was then stirred for 1h and stored at 4°C。

Sample Preparation for the Detection with Dark-Field Microscopy (DFM) and Scattering Spectroscopy. The AuNPs that were used for DFM images were fixed on the surface of the glass sheet. The glass sheet was sonicated in ethanol and then washed with pure water. The clean glass sheet was dried, and the AuNP solution was deposited on the surface to evaluate the DFM detection. To image cells that had absorbed AuNPs, the cell suspension (1 mL, 1×10^6 mL^{-1}) was seeded in each culture dish (the bottom is glass sheet) and cultured overnight. Then, 30 μL of the AuNP-60/APBA was added to each dish and incubated for various times at 37 °C. Furthermore, the dishes were washed twice and immersed in PBS for DFM imaging. Then, 50 μL of the AuNP-wrapped mannan was added to each dish. The AuNP-scattered light was split using a grating. And the scattering spectra of the samples were recorded by CCD spectrometer.

NIR Laser-Induced Heat Conversion. For measuring the photothermal conversion performance of AuNP60/APBA-AuNP13/MN, the temperature changes with different concentrations (equivalent to 1 nM, 2 nM, 4 nM, 6 nM, 8 nM, 10 nM) of the probes and different times of NIR laser irradiation. The NIR irradiation time of 30 min was selected for the following measurements. NIR laser (680 nm) at a power density of 0.5 W/cm^2 was used to irradiate the solution. The temperature changes were recorded by temperature gauge.

Calculation of the Photothermal Conversion Efficiency. The photothermal conversion efficiency of AuNP60/APBA-AuNP13/MN was determined according to previous method. [S1-S3] The detailed calculation was given as following:

Based on the total energy balance for this system:

$$\sum_i m_i C_{p,i} \frac{dT}{dt} = Q_{NPs} + Q_s - Q_{loss} \quad (1)$$

where m and C_p are the mass and heat capacity of solvent (water), respectively. T is the solution

temperature.

Q_{NPs} is the photothermal energy input by AuNP60/APBA-AuNP13/MN:

$$Q_{NPs} = I(1 - 10^{-A\lambda})\eta \quad (2)$$

Where I is the laser power, A_λ is the absorbance of AuNP60/APBA-AuNP13/MN at the wavelength of 680 nm, and η is the conversion efficiency from the absorbed light energy to thermal energy.

Q_s is the heat associated with the light absorbance of the solvent, which is measured using pure water without AuNP60/APBA-AuNP13/MN irradiation with 680 nm laser at the power densities of 0.5 W/cm^2.

Q_{loss} is thermal energy lost to the surrounding:

$$Q_{loss} = hA\Delta T \quad (3)$$

Where h is the heat transfer coefficient, A is the surface area of the container, and ΔT is the temperature change, which is defined as $T-T_{surr}$ (T and T_{surr} are the solution temperature and ambient temperature of the surroundings, respectively).

At the maximum steady-state temperature, the heat input is equal to the heat output, that is:

$$Q_{NPs} + Q_s = Q_{loss} = hA\Delta T_{max} \quad (4)$$

Where ΔT_{max} is the temperature change at the maximum steady-state temperature. According to the Eq.2 and Eq.4, the photothermal conversion efficiency (η) can be determinded:

$$\eta = \frac{hA\Delta T_{max} - Q_s}{I(1-10^{-A\lambda})} \quad (5)$$

In this equation, only hA is unknown for calculation. In order to get hA, we here in introduce θ, which is defined as the ratio of ΔT to ΔT_{max}:

$$\theta = \frac{\Delta T}{\Delta T_{max}} \quad (6)$$

Substituting Eq.6 into Eq.1 and rearranging Eq.1:

$$\frac{d\theta}{dt} = \frac{hA}{\sum_i m_i C_{p,i}}\left[\frac{Q_{NPs}+Q_s}{hA\Delta T_{max}} - \theta\right] \quad (7)$$

When the laser was shut off, the $Q_{NPs} + Q_s = 0$, Eq.7 changed to:

$$dt = -\frac{\sum_i m_i C_{p,i,d\theta}}{hA \quad \theta} \quad (8)$$

Integrating Eq.8 gives the expression:

$$t = -\frac{\sum_i m_i C_{p,i}}{hA}\theta \quad (9)$$

Thus, hA can be determined by applying the linear time data from the cooling period vs-ln θ. Substituting hA value into Eq.5, the photothermal conversion efficiency (η) of AuNP60/APBA-AuNP13/MN can be calculated.

Cell Culture. MCF-7 cells were cultured in Dulbecco's modified Eagle's medium (DMEM, GIBCO, Sigma-Aldrich Inc., USA), which was supplemented with streptomycin (100 μg· mL^{-1}), fetal bovine serum (10%, FBS, Sigma), and penicillin (100 μg· mL^{-1}) at 37 °C in an environment with 5% CO_2. The cell number was determined with a cell-counting board.

Detection of p53 (WTp53, Total p53, and MTp53) in Cell Extracts. In total, 4×10^7 cells were collected, washed twice with cold PBS solution (0.1 M, pH 7.4), and resuspended in lysis buffer (200 μL). The lysis buffer contained 50 mM Tris-HCl (pH 8.0), 1 mM $MgCl_2$, 0.1 mM PMSF, 1 mM EGTA, 10% glycerol, and 0.5% CHAPS. The mixture was incubated on ice for 30 min and sonicated for 1 min. After centrifugation at 16000 rpm and 4 °C for 20 min, the upper clear liquid was collected and diluted to 200 μL as the cell extract. The standard curve method was used to quantify the MTp53 in the MCF-7 cell extract. As mentioned above, to measure the MTp53 in cell extracts, aggregation of 30 μL AuNP-60/APBA with 50μL AuNP-13/MN was added to cells. The solution was treated with a series of standard MTp53 fluorescent protein at different final concentrations, and relative fluorescence value was obtained and calculated. To measure the total p53 (WTp53 and MTp53) in cell extracts, a p53 pan ELISA kit which was performed on a microplate reader (Fluostar Omega, BMG Labtech, Germany). The total p53 amount in the sample was calculated using a standard calibration curve that was established using the standards contained in the kit. Thus, by subtracting the amounts of total p53 and WTp53, the amount of MTp53 was obtained. The experiment was performed at least three times to check the reproducibility and obtain the average and standard deviation values prior to illustrate in the graph.

Cytotoxicity of the gold nanoparticle bouquet. The cytotoxicity of the proposed probe was tested with MCF-7 cells by MTT assay. 1.0×10^4 MCF-7 cells were planted into 96-multiwell plates, and incubated overnight at 37℃ in a humidified atmosphere of 5% CO_2. Probes were diluted to different concentrations with culture medium (equivalent to 1 nM, 2 nM, 4 nM, 6 nM, 8 nM, 10 nM). The cells were incubated with these probe solutions (100 μL) for 12 h at 37℃ under 5% CO_2. The cells were washed with PBS and with culture medium (100 μL) before being illuminated at ambient temperature. Then, the cells were illuminated with the laser (680 nm) for 20 min at 0.5W cm^{-2} at 37℃. The tumor cell apoptosis process was observed under a DFM/fluorescence microscope. After MTT solution (50 μL, 1 mg mL^{-1}) was added to each well and incubated for 4 h at 37 °C, dimethyl sulphoxide (100 μL) was added to each well and the plate was silenced for 15 min at 37 °C. The absorbance at 450 nm was detected to calculate the cell viability (%) by $(A_{test}/A_{control})\times100\%$. The experiment was performed at

least three times to check the reproducibility and obtain the average and standard deviation values prior to illustrate in the graph.

EdU staining. Cell proliferation was further determined by incorporation of 5-ethynyl-20-deoxyuridine (EdU) with the EdU Cell Proliferation Assay Kit (RiboBio, China). Briefly, 4×10^4 MCF-7 cells were seeded per well in a 24 well plate and left to adhere for 12 h. Cell media containing 10 nM AuNP60/APBA-AuNP13/MN nanoparticle bouquet or solvent control were added and incubate for 12 h followed by illumination for 20 minutes. After another 12 h, cell media were changed, and fresh media containing 50 μM EdU were added. After 4 hours incubation, cells were washed twice with PBS and fixed using 4% (w/v) paraformaldehyde in PBS. The later procedures were performed according to the manufacturer's protocol. Image acquisition was achieved on the EVOS Cell Imaging Systems (Life Technologies, USA). Image acquisition was automated, and 8-10 frames were taken per well. Images were analyzed using the ImageJ Software (Ver1.6, NIH, USA). The experiment was performed at least three times to check the reproducibility and obtain the average and standard deviation values prior to illustrate in the graph.

TUNEL assay. Cell apoptosis was detected by TUNEL FITC Apoptosis Detection Kit (Vazyme, China). In brief, 1×10^5 MCF-7 cells were seeded per well in a 24 well plate and left to adhere for 12 h. Cell media containing 10 nM AuNP60/APBA-AuNP13/MN nanoparticle bouquet or solvent control were added and incubated for 12 h followed by illumination for 20 min. After another 12 h, cells were washed twice with PBS and fixed using 4% (w/v) paraformaldehyde in PBS, then performed according to the manufacturer's protocol. Image acquisition was achieved on the EVOS Cell Imaging Systems. Image acquisition was automated, and 6-8 frames were taken per well. Images were analyzed using the Image J Software. The experiment was performed at least three times to check the reproducibility and obtain the average and standard deviation values prior to illustrate in the graph.

Monitoring Photothermal Therapy in Living Mice. The mice (5- to 6-week old male BALB/C mice) were acquired from the Animal Core Facility of Nanjing Medical University (Nanjing, China). All experiments on animals were implemented to conform to the Animal Care and Use Committee of Nanjing Medical University (no. IACUC-1807022-3). Animals were converged at a dominated temperature (20±2°C) with a 12h light-dark cycle and unconstrained food and water. The MCF-7 cells (in the logarithmic growth phase) were injected subcutaneously on the flank of the nude mice under anesthesia with isoflurane to establish tumor model. Tumor volume was measured with a Vernier caliper (using 0.5×length×width). The binary system was then intravenously injected into tumor-bearing mice. After injection (24 h), the tumor region was irradiated with a 630 nm laser with a power of 0.5 W cm^{-2}

for 30 min for photothermal treatment. The therapeutic effect was evaluated by monitoring the tumor volume after the NIR treatment. The mice were euthanized after NIR treatment. And the tumor tissues and other organs were collected to carry on hematoxylin-eosin (H&E) staining for histological study (n=5 in each group). The mice without NIR treatment were used as the control group.

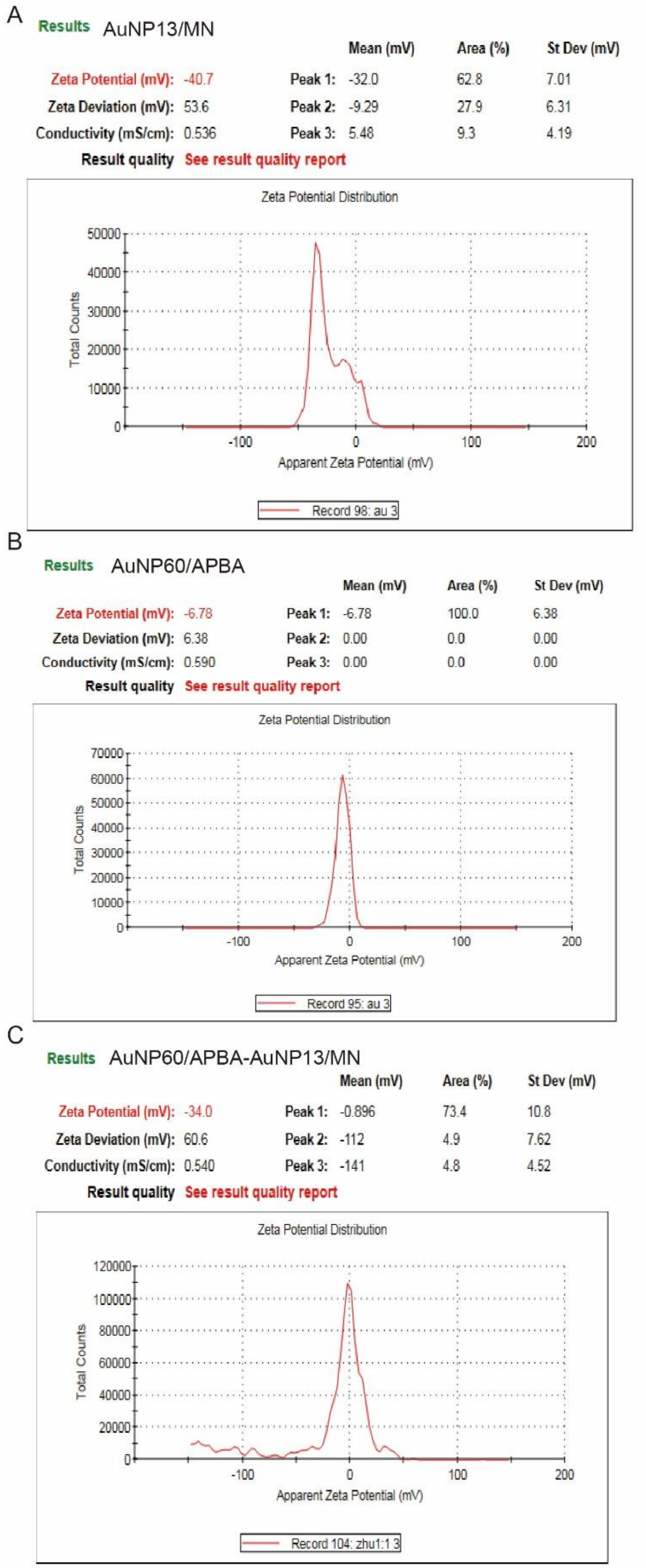

Figure S1. Zeta-potentials as-synthesized samples at different stages of (A) AuNP13/MN; (B) AuNP60/APBA and (C) AuNP60/APBA-AuNP13/MN.

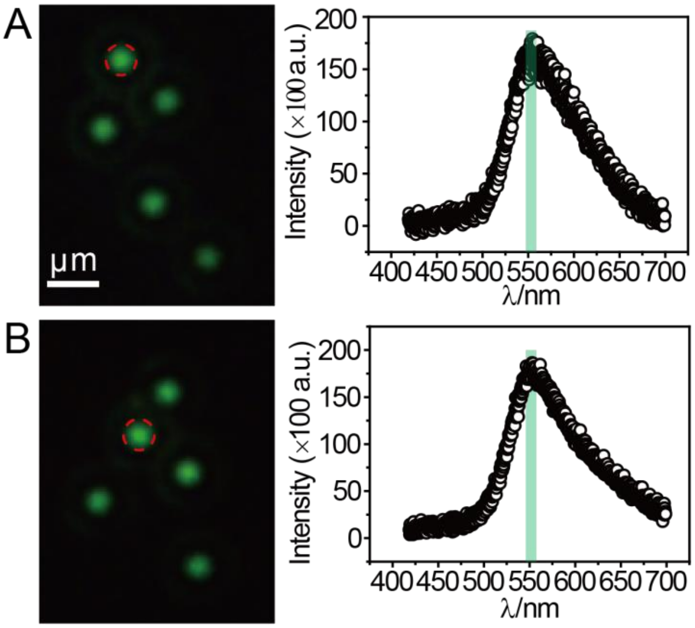

Figure S2. DMF images and scattering spectra of (A) 60 nm AuNP; (B) 60 nm AuNP surface modification APBA.

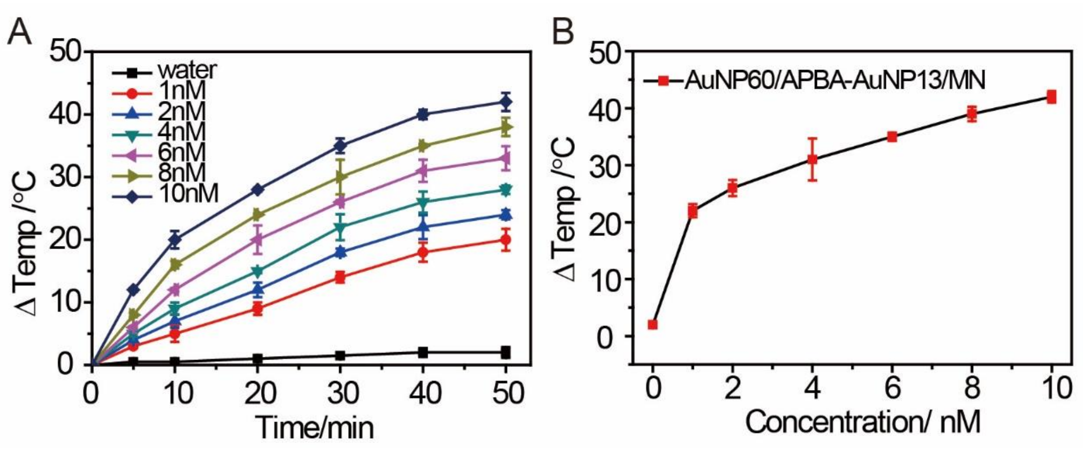

Figure S3. (A) Temperature increase of water and AuNP60/APBA-AuNP13/MN with different concentrations as a function of irradiation time. (B) Plot of temperature change over a period of 50 min versus the concentration of AuNP60/APBA-AuNP13/MN nanoparticle bouquet.

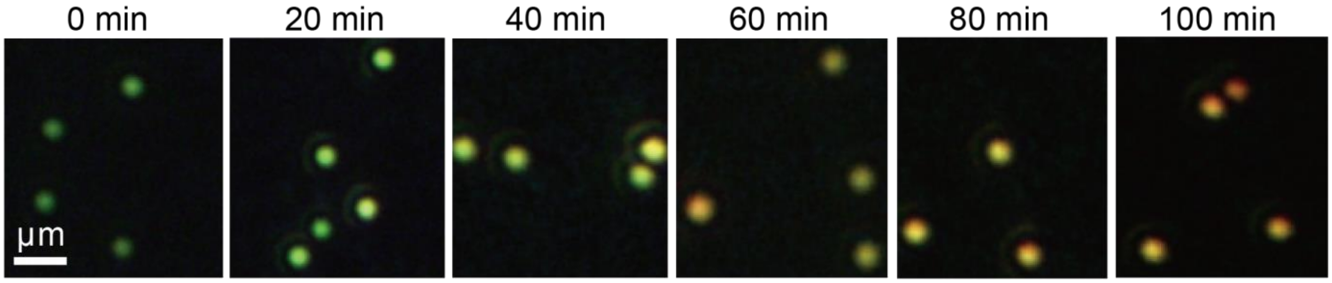

Figure S4. Time course of DFM images of the AuNP-60/APBA incubated with AuNP-13/MN.

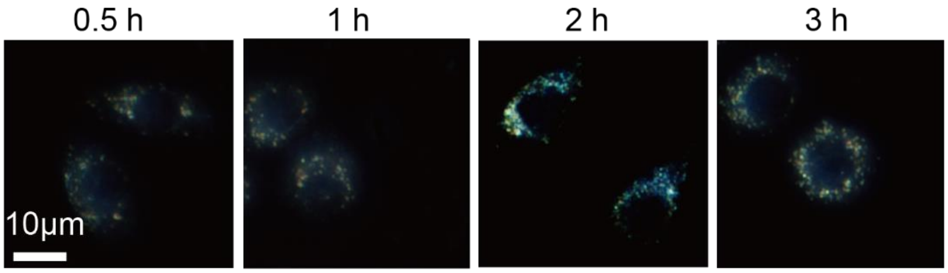

Figure S5.Time course of DFM images of the MCF-7 cells incubated with 30 µL AuNP-60/APBA incubated with 50µL AuNP-13/MN.

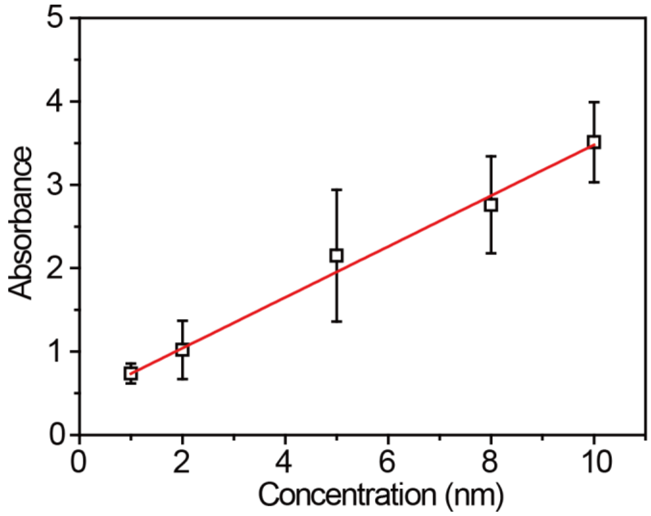

Figure S6. Plot of Absorbance value *vs*. total p53 concentration.

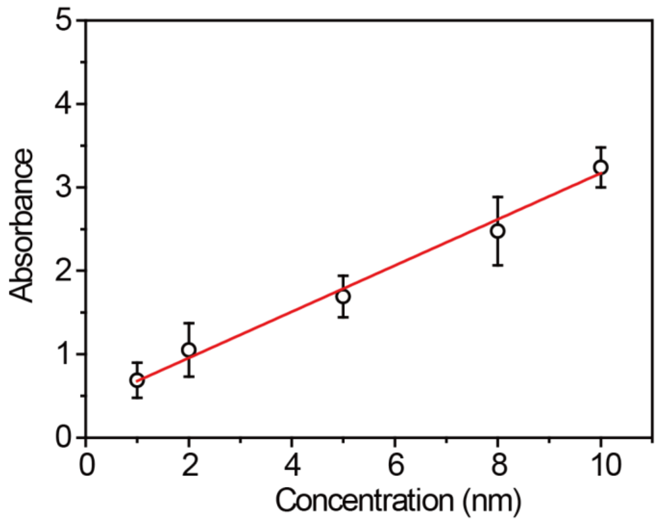

Figure S7. Plot of Absorbance value *vs*. MU p53 concentration.

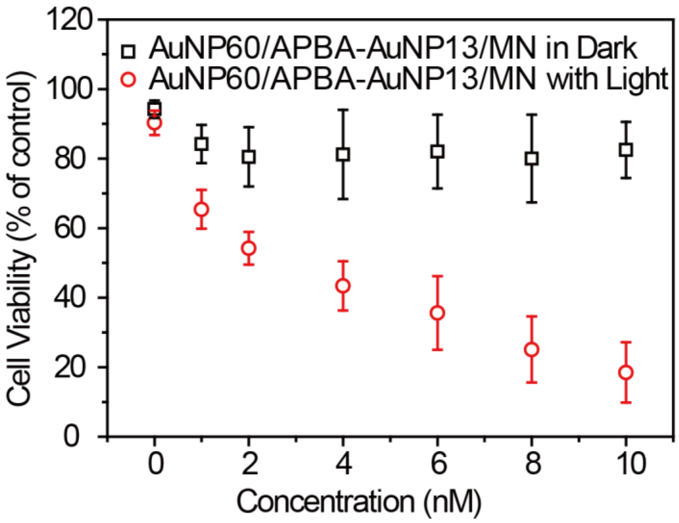

Figure S8. Viability of MCF-7 cells (100 μL, 1.0×10^{6} mL^{-1}) treated with 30 μL AuNP-60/APBA and AuNP-13/MN solution after different concentrations (equivalent to 1 nM, 2 nM, 4 nM, 6 nM, 8 nM, 10 nM).

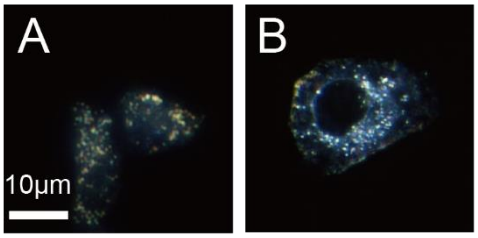

Figure S9. DFM images of (A) MCF-7 cells (B) after incubation with 30 μL AuNP-60/APBA and AuNP-13/MN solution after illumination of 20 min.

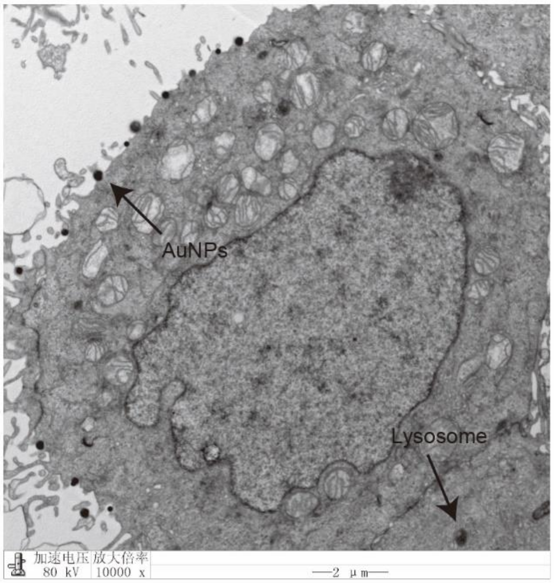

Figure S10. TEM images of MCF-7 cells incubated with AuNP-60/APBA and AuNP-13/MN (4 nM) solution for 2 h.

References:

1. Maye, M. M.; Nykypanchuk, D.; Van der Lelie, D.; Gang, O. A Simple Method for Kinetic Control of DNA-Induced Nanoparticle Assembly. *J. Am. Chem. Soc.* **2006**, 128, 14020.
2. Lee, P. C.; Meisel, D. Adsorption and surface-enhanced Raman of dyes on silver and gold sols. *The Journal of Physical Chemistry*, **1982**, 86(17): 3391-3395.
